# Correction: Amin et al. Evaluation of Medication Prescribing Applications Available in Australia. *Pharmacy* 2023, *11*, 49

**DOI:** 10.3390/pharmacy11050165

**Published:** 2023-10-13

**Authors:** Riya Amin, Melissa Cato, Sasha Rahavi, Kristin Tran, Kenneth Lee, Elton Lobo, Deanna Mill, Amy Page, Sandra Salter

**Affiliations:** 1 Pharmacy, School of Allied Health, The University of Western Australia, Crawley, WA 6009, Australiakenneth.lee@uwa.edu.au (K.L.);; 2 Department of General Practice, The University of Melbourne, Melbourne, VIC 3010, Australia; 3 Institute for Physical Activity and Nutrition (IPAN), Deakin University, Geelong, VIC 3220, Australia

**The authors wish to make the following corrections to this paper [1]:**

## 1. Change to Title

Based on external feedback provided post-acceptance, the title was updated to present a more neutral tone: **Evaluation of Medication Prescribing Applications Available in Australia**. The original title was, ‘**Is It Safe to Buy Medications from an App? Evaluation of Medication Prescribing Applications Available in Australia**’.

## 2. Text Correction

Upon revaluation, after concerns from a reader were raised, various changes to the text were made to improve clarity. Key changes were made to the identification of which researcher performed which aspect of the research (see Sections 2.1.3, 2.1.4, 2.2.1 and 2.2.2). Additionally, the strengths and limitations and future research sections were elaborated to provide a more robust discussion.

All of the changes are provided below for transparency:

**(Section 1)** The growth in digital health strategies has seen an increase in the availability of eHealth web apps for consumers [1,2]. One category of eHealth apps that has emerged recently is prescription request apps. These apps allow consumers to request prescriptions for specific prescription-only medications.

**(Section 2.1.3)** For each search term across the 3 platforms, the first 50 results, excluding advertisements, were recorded in Microsoft Excel (Microsoft Corporation, Redmond, Washington USA; version 16.64). All results were collated, duplicates were removed, and results were screened independently by two researchers (RA and SR) according to the exclusion criteria. All iOS App Store and Google Play results were screened using the app store’s description (e.g., title, images, or text). Each Google search engine result was accessed, and the web’s homepage was reviewed to identify and exclude results that were not web apps.

The identified mobile apps were downloaded. Any discrepancies in app inclusion were resolved through discussion.

**(Section 2.1.4)** Two researchers (KT and MC) individually accessed each app and extracted information on app features in an iterative process.

**(Section 2.2.1)** The data extraction phase demonstrated that the apps typically use a questionnaire to facilitate the consultation and prescribing process for each medication. Two researchers (KT and MC) explored the self-select prescription function of each prescribing app to identify the questions posed when a consumer requests a prescription.

**(Section 2.2.2)** The lists of criteria were developed by two researchers (KT and MC). To minimize bias, each app was then assessed independently by two other researchers (RA and SR), results were compared, and any discrepancies were resolved through discussion.

These competencies were considered to have been met if the app satisfied at least 50% of the developed criteria for each medication; this 50% criterion was determined by the team given that 50% in assessments typically constitutes a ‘pass’ and to facilitate consistency in decision making.

**(Section 3.2)** None of the apps met all competencies for any medication explored in this study. Apps B (when prescribing fluticasone/salmeterol and colchicine) and F (when prescribing Levlen^®^) were the most adherent to the NPS framework and met eight of the competencies, as shown in Table 5.

**(Section 4.1)** Our study has notable limitations that are important to discuss. No prescriptions were obtained as the researchers could not procure the medications using the identification required by the apps. This meant that we were unable to assess what happens after responses to the questionnaires are submitted for review by the health professional; as such, our study was not able to determine whether a consumer’s request may lead to a referral to a consultation with a health professional, and therefore we cannot definitively conclude the safety of procuring medications from an app. Further, we selected only five medications advertised on the apps and used a standardized consumer to represent a cross-section of considerations that must be made when prescribing and examined the prescribing process in detail. However, the prescription request apps may have performed differently had we chosen different medications or consumer parameters. Similarly, considering the selective nature of this study, the apps may have performed differently had we chosen to assess all advertised medications. Further, the mock consumer profile that we created in Box 1 depicts consumers who are healthy and do not have co-morbidities; thus, we did not determine whether creating a mock consumer profile with significant co-morbidities would have yielded different results. Finally, there does not appear to be a unified framework for assessing prescribing competencies; as such, there may be more appropriate frameworks than the NPS 12-core competencies we have used. However, our study highlights some potential gaps in the identified apps.

**(Section 4.2)** There are no known studies investigating the quality and appropriateness of prescribing services offered by prescription request apps in parallel to traditional face-to-face doctor consultations. Additionally, as we only focused on prescription request apps that do not involve video/verbal telehealth consultations, it is unknown how apps that utilize this functionality would fare against prescribing competencies.

The authors state that the scientific conclusions are unaffected. This correction was approved by the Academic Editor. The original publication has also been updated.
