# Supplementary material for: Evaluation of Medication Prescribing Applications Available in Australia
Source: Pharmacy (Basel). 2023 Mar 6;11(2):49. doi: 10.3390/pharmacy11020049 (PMC10037640; doi:10.3390/pharmacy11020049)
Supplement: Supplementary file 1 [file pharmacy-11-00049-s001.zip › Supplementary - clean.pdf]

Table S1. Medication 1 (Levlen®), selected to assess prescribing app adherence to the NPS '12 core competencies for safe prescribing'. Apps that were considered to have addressed the competency must score 50% or above. For the following competencies: Medical History, Medication History, Contraindications, Dose Regimen and Treatment Monitoring, the apps had to fulfil at least half of the developed criteria before it could be considered to have addressed the competency.

**Competencies**	**App A**	**App B**	**App C**	**App D**	**App E**	**App F**	**App G**
**Medical History**	45%	91%	82%	73%	55%	82%	82%
Allergies	0	1	1	1	1	1	1
Age	1	1	1	1	1	1	1
BMI	0	1	1	1	0	1	1
Alcohol	0	1	1	0	0	1	0
Breastfeeding	1	1	1	1	1	1	1
Pregnant	1	1	1	1	1	1	1
Exercise	0	1	0	0	0	0	0
Gender	1	1	1	0	1	1	1
Transgender	0	0	0	1	0	0	1
Medical conditions	0	1	1	1	0	1	1
Smoking	1	1	1	1	1	1	1
**Medication History**	0%	100%	50%	100%	0%	100%	100%
OTC/CAM	0	1	0	1	0	1	1
Other prescription medications	0	1	1	1	0	1	1
**Further Investigation**	0	0	0	0	0	0	0
**Adherence**	0	0	0	0	0	0	0
**Shared Decision Making**	0	0	0	0	0	0	0
**Disease Management**	1	1	1	1	1	1	1
**Indication**	0	1	0	0	0	1	1
**Other Treatment**	0	0	0	0	0	1	0
**Contraindications**	64%	86%	71%	50%	64%	79%	79%
Migraines	1	1	1	1	1	1	1
Vaginal bleeding	1	1	1	1	1	0	0
Breast cancer	1	1	1	1	1	1	1
Current or history of VTE	1	1	1	1	1	1	1
Postpartum	0	0	0	0	0	1	1
Current/History of CAD or stroke	1	1	1	0	1	0	0
Congenital heart disease or complicated vasculature	0	1	0	0	0	1	1
Surgery	0	1	0	0	0	1	1
Hepatic impairment/Liver problems	1	1	1	1	1	1	1
Clotting problems (APLAs, thrombogenic mutation)	0	1	1	0	0	1	1
Pancreatitis	1	1	1	0	1	0	0
Gynaecological cancer	0	1	1	0	0	1	1
Severe hypertension	1	1	1	1	1	1	1
Antivirals	1	0	0	1	1	1	1
**Dose Regimen**	50%	100%	50%	50%	50%	100%	100%
Dose/duration	0	1	0	0	0	1	1
Current medication	1	1	1	1	1	1	1
**Communicate (outpatient)**	0	0	0	0	0	0	0
**Treatment Monitoring**	0	100%	100%	100%	50%	50%	50%
Side effects	0	1	1	1	0	1	1
Referral	0	1	1	1	1	0	0

Key*: 0 = criteria not fulfilled; 1 = criteria fulfilled*

Table S2. Medication 2 (fluticasone/salmeterol), selected to assess prescribing app adherence to the NPS '12 core competencies for safe prescribing'. Apps that were considered to have addressed the competency must score 50% or above. For the following competencies: Medical History, Medication History, Dose Regimen and Treatment Monitoring, the apps had to fulfil at least half of the developed criteria before it could be considered to have addressed the competency.

Competencies	App A	App B	App C	App E	App F
**Medical History**	50%	80%	70%	50%	70%
Allergies	1	1	1	1	1
Age	1	1	1	1	1
BMI	0	1	1	0	1
Alcohol	0	1	1	0	1
Breastfeeding	1	0	0	1	0
Pregnant	1	1	0	1	0
Exercise	0	0	0	0	0
Gender	1	1	1	1	1
Medical conditions	0	1	1	0	1
Smoking	0	1	1	0	1
**Medication History**	0%	100%	50%	0%	50%
OTC/CAMs	0	1	0	0	0
Other prescription medications	0	1	1	0	1
**Further Investigations**	0	0	0	0	0
**Adherence**	0	0	0	0	0
**Shared Decision Making**	0	0	0	0	0
**Disease Management**	1	1	0	0	1
**Indication**	0	1	1	1	1
**Other Treatment**	1	1	1	0	1
**Contraindications**	N/A	N/A	N/A	N/A	N/A
**Dose Regimen**	50%	100%	100%	50%	0%
Dose/duration	0	1	1	0	0
Current medication	1	1	1	1	0
**Communicate (outpatient)**	0	0	0	0	0
**Treatment Monitoring**	50%	100%	50%	50%	50%
Referral	1	1	1	0	1
Side effects	0	1	0	1	0

Key: *0 = criteria not fulfilled; 1 = criteria fulfilled*

Table S3. Medication 3 (sildenafil), selected to access prescribing app adherence to the NPS '12 core competencies for safe prescribing'. Apps that were considered to have addressed the competency must score 50% or above. For the following competencies: Medical History, Medication History, Contraindications, Dose Regimen and Treatment Monitoring, the apps had to fulfil at least half of the developed criteria before it could be considered to have addressed the competency.

Competencies	App A	App B	App C	App D	App E
**Medical History**	33%	89%	78%	100%	44%
Allergies	0	1	1	1	1
Age	1	1	1	1	1
BMI	0	1	1	1	0
Alcohol	0	1	1	1	0
Exercise	0	1	0	1	0
Gender	1	1	1	1	1
Transgender	1	0	0	1	1
Medical conditions	0	1	1	1	0
Smoking	0	1	1	1	0
**Medication History**	0%	100%	50%	100%	0%
OTC/CAMs	0	1	0	1	0
Other prescription medications	0	1	1	1	0
**Further Investigation**	0	0	0	0	0
**Adherence**	0	0	0	0	0
**Shared Decision Making**	0	0	0	0	0
**Disease Management**	0	0	1	1	0
**Indication**	0	0	1	1	0
**Other Treatment**	0	0	0	0	0
**Contraindications**	45%	100%	27%	91%	73%
Nitrates/Nitrites	1	1	0	1	1
GCS (riociguat)	1	1	0	0	1
Hypotension	0	1	1	1	0
Hypertension	0	1	1	1	1
Recent MI	1	1	0	1	1
Recent stroke	0	1	0	1	1
Other CVD	0	1	1	1	1
Exercise tolerance	0	1	0	1	0
Severe hepatic impairment	0	1	0	1	0
History of NAION	1	1	0	1	1
Hereditary degenerative retinal disorders	1	1	0	1	1
**Dose Regimen**	0%	75%	25%	38%	13%
Dose/duration	0	1	1	0	0
Current medication	0	1	1	0	1
Medical conditions requiring dose adjustment	0	0	0	1	0
Erythromycin	0	0	0	0	0
Antivirals	0	1	0	0	0
Azoles	0	1	0	0	0
Liver impairment	0	1	0	1	0
Kidney function	0	1	0	1	0
**Communicate (outpatient)**	0	0	0	0	0
**Treatment Monitoring**	0%	50%	50%	0%	50%
Side effects	0	1	0	0	0
Referral	0	0	1	0	1

***Key**: 0 = criteria not fulfilled; 1 = criteria fulfilled*

Table S4. Medication 5 (sertraline), selected to assess prescribing app adherence to the NPS '12 core competencies for safe prescribing'. Apps that were considered to have addressed the competency must score 50% or above. For the following competencies: Medical History, Medication History, Contraindications, Dose Regimen and Treatment Monitoring, the apps had to fulfil at least half of the developed criteria before it could be considered to have addressed the competency.

**Competencies**	**App A**	**App B**	**App E**
**Medical History**	40%	70%	50%
Allergies	0	1	1
Age	1	1	1
BMI	0	0	0
Alcohol	0	1	0
Breastfeeding	1	0	1
Pregnant	1	1	1
Exercise	0	0	0
Gender	1	1	1
Medical conditions	0	1	0
Smoking	0	1	0
**Medication History**	0%	100%	0%
OTC/CAMs	0	1	0
Other prescription medications	0	1	0
**Further Investigation**	0	0	0
**Adherence**	0	0	0
**Shared Decision Making**	0	0	0
**Disease Management**	1	1	0
**Indication**	1	1	0
**Other Treatment**	0	1	1
**Contraindications**	100%	0%	100%
MAOI or moclobemide	1	0	1
**Dose Regimen**	33%	100%	33%
Dose/Duration	0	1	0
Current medications	1	1	1
Hepatic impairment	0	1	0
**Communicate (outpatient)**	0	0	0
**Treatment Monitoring**	50%	100%	100%
Referral	1	1	1
Side effects	0	1	1

Key: *0 = criteria not fulfilled; 1 = criteria fulfilled*

Table S5. Medication 5 (colchicine), selected to assess prescribing app adherence to the NPS '12 core competencies for safe prescribing'. Apps that were considered to have addressed the competency must score 50% or above. For the following competencies: Medical History, Medication History, Contraindications, Dose Regimen and Treatment Monitoring, the apps had to fulfil at least half of the developed criteria before it could be considered to have addressed the competency.

**Competencies**	**App A**	**App B**	**App C**	**App D**	**App E**
**Medical History**	30%	80%	70%	50%	50%
Allergies	1	1	1	1	1
Age	1	1	1	1	1
BMI	0	1	1	0	0
Alcohol	0	1	1	0	0
Breastfeeding	0	0	0	1	1
Pregnant	0	1	0	1	1
Exercise	0	0	0	0	0
Gender	1	1	1	0	1
Medical conditions	0	1	1	1	0
Smoking	0	1	1	0	0
**Medication History**	0%	100%	100%	100%	0%
OTC/CAM	0	1	1	1	0
Other prescription medications	0	1	1	1	0
**Further Investigation**	0	0	0	0	0
**Adherence**	0	0	1	0	0
**Shared Decision Making**	0	0	0	0	0
**Disease Management**	0	1	0	0	0
**Indication**	1	1	0	1	0
**Other Treatment**	0	1	1	1	0
**Contraindications**	67%	67%	50%	100%	50%
Blood dyscrasis	1	1	0	1	1
Renal impairment	1	1	1	1	1
Liver impairment	1	1	1	1	1
P-gp/CYP meds	0	0	0	1	0
GIT	0	0	0	1	0
CVD	1	1	1	1	0
**Dose Regimen**	0%	100%	100%	0%	50%
Dose/Duration	0	1	1	0	0
Current medication	0	1	1	0	1
**Communicate (outpatient)**	0	0	0	0	0
**Treatment Monitoring**	0%	50%	50%	50%	50%
Side effects	0	1	0	0	1
Referral	0	0	1	1	0

***Key**: 0 = criteria not fulfilled; 1 = criteria fulfilled*
